# Early BK Polyomavirus–Specific Immunity and Viral Clearance

**DOI:** 10.1016/j.ekir.2026.107064

**Published:** 2026-08-31

**Authors:** Dominique Bertrand, Tristan De Nattes, Charlotte Laurent, Mathilde Lemoine, Ludivine Lebourg, Mélanie Hanoy, Frank Le Roy, Steven Grangé, Eleusis Mauger, Amandine Becquet, Marie Gueudin, Dominique Guerrot, Sophie Candon

**Affiliations:** 1 Department of Nephrology, Transplantation and Hemodialysis, Rouen University Hospital, Rouen, France; 2 Institut national de la santé et de la recherche médicale, INSERM U1234, University of Rouen Normandy, Rouen, France; 3 Department of Virology, Rouen University Hospital, Rouen, France; 4 Institut national de la santé et de la recherche médicale, INSERM DYNAMICURE UMR 1311, University of Rouen Normandy, Rouen, France; 5 Department of Immunology and Biotherapies, Rouen University Hospital, Rouen, France

**Keywords:** BK virus, ELISPOT, kidney transplantation, polyomavirus-associated nephropathy, T cells

## Abstract

**Introduction:**

BK polyomavirus (BKPyV) replication remains a frequent complication after kidney transplantation. The clinical relevance of assessing BKPyV-specific cellular immunity at the time of first detectable viremia remains incompletely defined.

**Methods:**

We retrospectively studied 50 kidney transplant recipients with a first episode of BKPyV DNAemia who underwent BKPyV-specific interferon-gamma enzyme-linked immunosorbent spot (IFN-γ ELISPOT) shortly after the first positive plasma polymerase chain reaction (PCR). Responses directed against structural (viral protein [VP]1–VP3) and regulatory (large T and small t) antigens were quantified. The primary end point was time from ELISPOT to viral clearance. Secondary end points included viral clearance within 90 days, biopsy-proven BKPyV-associated nephropathy (BKPyVAN), immunosuppression (IS) reduction, and kidney function outcomes.

**Results:**

Structural BKPyV-specific ELISPOT responses showed marked interindividual variability and correlated weakly with viral burden. Thirty-seven patients (74%) achieved viral clearance. Higher structural responses were independently associated with faster viral clearance (adjusted hazard ratio per 100 spot-forming cells [SFC]: 1.28; 95% confidence interval [CI]: 1.15–1.42; *P* < 0.0001), a lower incidence of biopsy-proven BKPyVAN, and a reduced need for advanced IS reduction (adjusted odds ratio [aOR] per 100 SFC: 0.34; 95% CI: 0.14–0.83; *P* = 0.018). Responses directed against regulatory antigens showed weaker and less consistent associations with outcomes.

**Conclusion:**

Early BKPyV-specific cellular immune responses measured by IFN-γ ELISPOT were consistently associated with viral clearance kinetics and clinically relevant outcomes after kidney transplantation. Structural antigen-specific responses may provide complementary information regarding antiviral immune competence and support further evaluation of immune-stratified management strategies. These findings should be considered hypothesis-generating and require prospective multicenter validation.

## Graphical abstract

**Figure:**
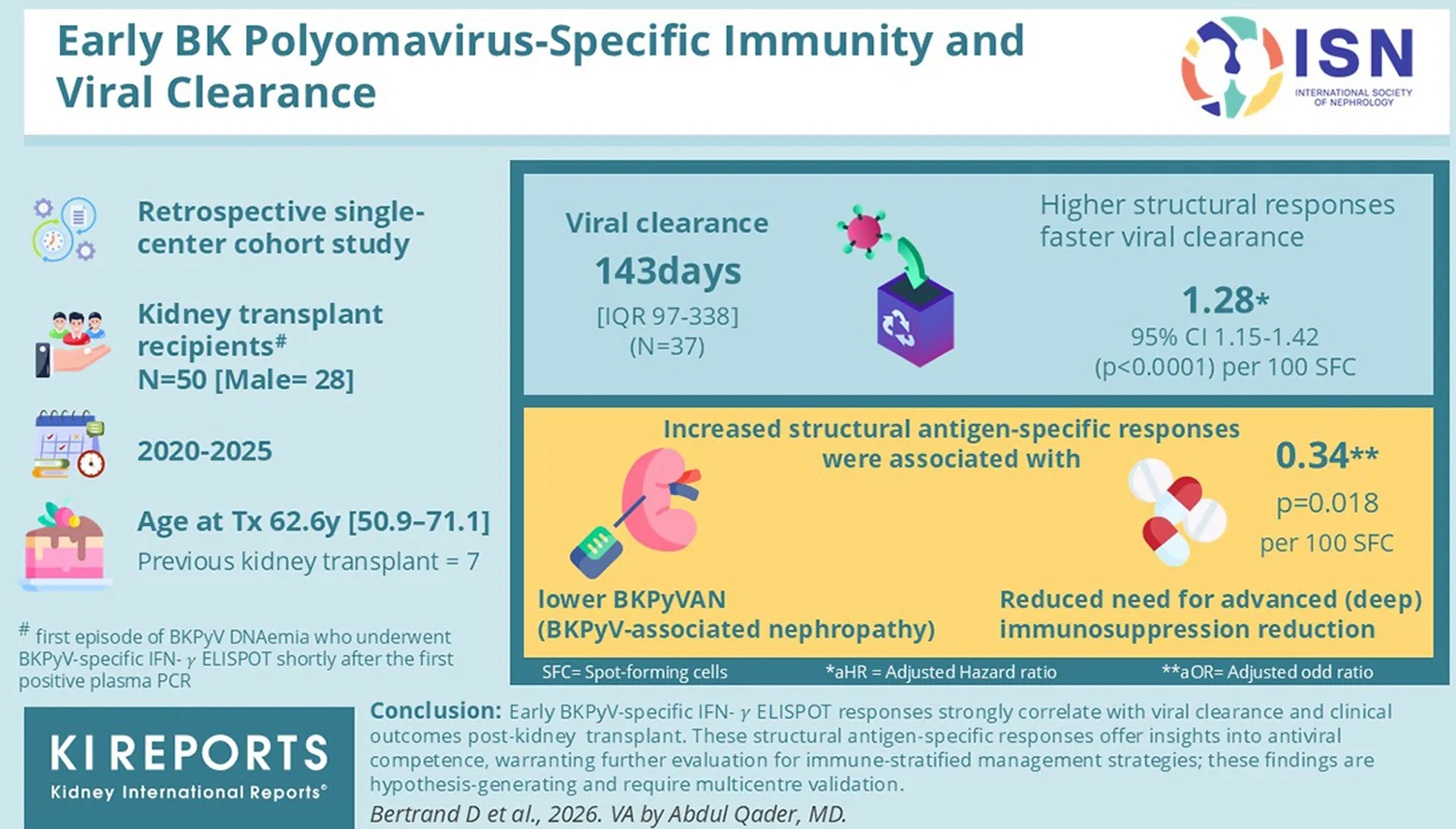

## Introduction

BKPyV replication remains 1 of the most frequent infectious complications after kidney transplantation and a major cause of potentially preventable graft injury through BKPyVAN.^1^, ^2^, ^3^, ^4^, ^5^ Prospective and registry studies show that BKPyV replication is frequent after kidney transplantation and that progression to BKPyVAN is associated with impaired graft survival.^2^^,^^4^^,^^6^ Importantly, the burden of BKPyV infection persists despite systematic screening, underscoring limitations of current management strategies.^7^, ^8^, ^9^

The current standard of care relies on regular monitoring of plasma BKPyV DNAemia and stepwise reduction of maintenance IS. This approach is now codified in the Second International Consensus Guidelines on the Management of BKPyV in Kidney Transplantation, published in 2024, which emphasize systematic screening and IS reduction as the cornerstone of therapy in the absence of proven antiviral agents.^7^ Earlier pivotal studies established that pre-emptive IS reduction can limit progression to BKPyVAN but at the cost of increased alloimmune risk, including acute rejection.^3^^,^^10^ Large cohort studies have shown that BKPyV replication follows heterogeneous trajectories after kidney transplantation and that persistent viremia and delayed viral clearance are associated with an increased risk of BKPyVAN and graft dysfunction.^6^^,^^8^^,^^11^

From a mechanistic perspective, control of BKPyV replication is primarily mediated by virus-specific cellular immunity. Early studies demonstrated that BKPyV-specific T-cell responses are suppressed by IS drugs and recover with IS reduction.^12^^,^^13^ Subsequent work showed that the presence and kinetics of BKPyV-specific IFN-γ–producing T cells are strongly associated with viral clearance and recovery from BKPyVAN.^13^, ^14^, ^15^, ^16^, ^17^ In this context, IFN-γ ELISPOT assays emerged as a practical tool to quantify BKPyV-specific cellular immunity in transplant recipients. However, the clinical value of assessing BKPyV-specific cellular immunity early after a first detectable viremia remains unclear.

We therefore conducted a single-center observational study in kidney transplant recipients with BKPyV DNAemia in whom BKPyV-specific IFN-γ ELISPOT was systematically performed shortly after the first positive plasma PCR. We aimed to determine whether early BKPyV-specific T-cell immunity is associated with viral clearance kinetics, the depth of IS reduction required during clinical management, the occurrence of biopsy-proven BKPyVAN, and subsequent kidney function evolution.

## Methods

### Study Design and Population

We conducted a retrospective single-center cohort study at a tertiary kidney transplant center. All adult kidney-only transplant recipients who met the following criteria were eligible:

• at least 1 first episode of plasma BK viremia above the local lower limit of quantification (LLOQ), and
• a BKPyV-specific interferon-γ ELISPOT performed after first viremia.

Patients with multiorgan transplantation were excluded.

Between 2020 and 2025, 50 consecutive eligible patients underwent BKPyV-specific ELISPOT testing after first BKPyV DNAemia. Data were extracted from electronic medical records and laboratory information systems. In accordance with French legislation, ethics committee approval was not required for this retrospective study using anonymized data. The study adhered to the principles of the Declaration of Helsinki and the Declaration of Istanbul.

### IS and BK Monitoring

Maintenance IS at our center was based on contemporary international recommendations and typically included the following: a calcineurin inhibitor (CNI, predominantly tacrolimus), mycophenolate mofetil (MMF) or mycophenolic acid, and corticosteroids, with basiliximab or lymphocyte-depleting induction according to immunological risk.

BKPyV monitoring followed international recommendations, with plasma PCR performed regularly during the first 2 post-transplant years (e.g., monthly in months 1–9, then every 3 months until month 24) and whenever clinically indicated.

Upon first BKPyV viremia, IS management followed routine practice and typically included initial MMF dose reduction. Treating clinicians were not informed of ELISPOT results during IS management. Plasma BKPyV DNAemia was measured by real-time quantitative PCR (AltoStar BK Virus PCR Kit 1.5, Altona Diagnostics, Hamburg, Germany) on a CFX96 platform (Bio-Rad, Hercules, CA), with a lower limit of quantification of 200 IU/ml.

Subsequent BKPyV management followed a stepwise protocol.

### BKPyV-Specific ELISPOT Assay

BKPyV-specific IFNγ-producing cellular responses were assessed at a dedicated recall visit scheduled approximately 2 to 4 weeks after first BK viremia, at which time plasma BK viral load was repeated.

Peripheral blood mononuclear cells were isolated by density gradient centrifugation and plated in triplicate at 2 × 10^5^ CD3+ T cells/well on anti–interferon-γ–coated ELISPOT plates (U-Cytech Biosciences, Utrecht, The Netherlands). Cells were stimulated with overlapping peptide pools corresponding to the following 5 BKPyV antigens: VP1, VP2, VP3 (structural capsid proteins), large T antigen, and small t antigen (JPT Peptide Technologies, Berlin, Germany). Negative control wells contained medium alone (Adoptive Immunotherapy Medium version 5; Thermo Fisher Scientific, Waltham, MA); positive control wells were stimulated with a commercial pool of 176 known peptide epitopes for a broad range of human leukocyte antigen subtypes and different infectious agents (CEFX Ultra SuperStim pool, JPT Peptide Technologies, Berlin, Germany).

After incubation (e.g., 16–20 hours), plates were developed according to the manufacturer’s instructions (U-Cytech Biosciences) and spots were counted using an automated reader (iSpot Spectrum, Autoimmun Diagnostika, Strassberg, Germany). Spot counts in antigen-stimulated wells were expressed as SFC per 10^6^ peripheral blood mononuclear cells after subtraction of background response in negative control wells.

All ELISPOT assays were performed using a standardized and analytically validated IFNγ ELISPOT platform demonstrating good intermediate precision across repeated measurements of antigen-specific responses.

For analysis, we defined 2 aggregated ELISPOT scores as follows:

• a structural ELISPOT score (BKV_struct): sum of SFC in response to VP1 + VP2 + VP3,
• a regulatory ELISPOT score (BKV_reg): sum of SFC in response to LT + ST.

The primary exposure was BKV_struct, analyzed as a continuous variable and scaled by 100 SFC increments (BKV_struct_100 = BKV_struct / 100) for interpretation.

### IS at the Time of ELISPOT

At ELISPOT assessment, the maintenance IS regimen, tacrolimus trough level, and MMF dose were recorded.

IS intensity at ELISPOT (IS_ELI) was estimated by grouping agents into the following 3 classes: corticosteroids, mycophenolate derivatives, and CNI-like agents (tacrolimus, everolimus, or belatacept).

We then counted the number of drug classes present at ELISPOT, and for multivariable models, IS_ELI was coded as a binary variable:

• triple therapy: all 3 classes present, IS_ELI = 0
• dual therapy: 2 classes present, IS_ELI = 1

Tacrolimus trough level and MMF dose were additionally evaluated as continuous covariates in sensitivity analyses.

### Outcomes

#### Primary End Point: Time From ELISPOT to Viral Clearance

Viral clearance was defined as 2 consecutive plasma BK PCR values below the lower limit of quantification. Time-to-event analyses used the ELISPOT date as the time origin. Patients without clearance were censored at the last available BK PCR or at graft loss, whichever occurred first.

#### Secondary End Points

1. *Early Viral Clearance.* For exploratory analyses, we defined early clearance as attainment of viral clearance within 90 days of the ELISPOT.
2. *Biopsy-Proven BK Virus Nephropathy.* BKPyVAN was defined by characteristic histologic lesions and positive SV40 large T-antigen immunohistochemistry on kidney allograft biopsy. Biopsies were clinically indicated and not protocol-driven; accordingly, analyses refer to biopsy-proven BKPyVAN rather than systematic exclusion of BKPyVAN in all patients.
3. *Depth of IS Reduction.* For each patient, the maximum intensity of IS reduction implemented during the BKPyV episode was reconstructed.

An ordinal IS reduction score (IS_stage) was defined as follows:

• stage 0: no reduction,
• stage 1: MMF or CNI dose reduction and/or mammalian target of rapamycin switch without drug discontinuation,
• stage 2: MMF discontinuation (without CNI switch and/or withdrawal),
• stage 3: MMF discontinuation followed by tacrolimus discontinuation with conversion to cyclosporine,
• stage 4: CNI (or belatacept) withdrawal with corticosteroid monotherapy.

For secondary analyses, deep IS reduction was defined as IS_stage ≥ 3 versus IS_stage ≤ 2.

4. *Additional Virological, Immunological, and Biological Assessments.* For each BK episode, we recorded the maximum plasma BK viral load (CV_max, copies/ml) observed from the first positive PCR to the end of follow-up. At the time of BK ELISPOT, total lymphocyte count and CD4/CD8 T-cell subsets were measured in peripheral blood using standard flow cytometry. Kidney function was assessed by serum creatinine at ELISPOT and at the end of follow-up, defined as the time of two consecutive negative BK PCR results or the last available measurement in patients without viral clearance. The change in creatinine (Δcreatinine = creatinine_end − creatinine_ELISPOT) was used as a secondary kidney outcome.

### Covariates

In addition to BK ELISPOT results and IS variables, we collected the following: recipient age and gender, primary kidney disease, number of previous transplants, time from transplantation to first BK viremia, time from first BK viremia to ELISPOT, plasma BK viral load at ELISPOT, expressed as log_10_ copies/ml (log10_CV_elispot), serum creatinine at ELISPOT.

### Statistical Analysis

Continuous variables are presented as median [interquartile range] and categorical variables as counts (percentages). Between-group comparisons used Mann–Whitney or Kruskal–Wallis tests for continuous variables and χ^2^ or Fisher’s exact tests for categorical variables, as appropriate. Time from ELISPOT to viral clearance was analyzed using Cox proportional hazards regression models adjusted for BKV_struct_100, log10_CV_elispot, IS_ELI, age, and time from transplantation to first BKPyV DNAemia. Logistic regression models were used for secondary outcomes, including deep IS reduction and biopsy-proven BKPyVAN. Because of the limited number of events, multivariable models included only a small number of clinically relevant covariates to reduce the risk of model overfitting.

Proportional hazards assumptions were checked using Schoenfeld residuals and log-minus-log plots. Sensitivity analyses additionally adjusted for tacrolimus trough levels and MMF dose at ELISPOT.

Receiver operating characteristic (ROC) analyses explored potential BKV_struct thresholds for early viral clearance (< 90 days), avoidance of deep IS reduction, and absence of biopsy-proven BKPyVAN.

Analyses were performed using R (version 4.3.3; R Foundation, Vienna, Austria). All tests were 2-sided, with *P* < 0.05 considered statistically significant.

## Results

### Patient Characteristics

During the study period, 568 kidney transplantations were performed at our center, among which 95 patients developed BKPyV DNAemia. This study included all consecutive patients who underwent BKPyV-specific ELISPOT testing after first BKPyV DNAemia and met the study eligibility criteria, resulting in a final cohort of 50 patients. Median age at transplantation was 62.6 years (IQR 50.9–71.1), and 28 (56%) were male, and 7 patients (14%) had received a previous kidney transplant (Table 1).

**Table 1:** Baseline characteristics of the 50 kidney transplant recipients at the time of BK ELISPOT

Characteristic	Overall (*N* = 50)
Age at transplant, yrs	62.6 (50.9–71.1)
Male gender, *n* (%)	28 (56)
Primary kidney disease, *n* (%)	
Glomerulonephritis (other than IgA)	14 (28)
Genetic nephropathy	10 (20)
IgA nephropathy	8 (16)
Vascular nephropathy	6 (12)
Tubulointerstitial/urologic nephropathy	6 (12)
Undetermined	5 (10)
Drug-induced nephropathy	1 (2)
Previous kidney transplant, *n* (%)	7 (14)
cPRA, %	0.0 (0.0–49.5)
Induction therapy, *n* (%)	
Basiliximab	26 (52)
Thymoglobulin	24 (48)
Time from transplant to first BK viremia, mos	3.0 (1.9–4.0)
Time from first BK viremia to ELISPOT, days	17.4 (11.1–35.2)
BK viral load at ELISPOT, log_10_ copies/ml	3.86 (3.46–4.50)
Maximum BK viral load during episode, log_10_ copies/ml	4.63 (4.01–5.27)
Serum creatinine at ELISPOT, μmol/l	139.5 (109.0–174.0)
Maintenance IS at ELISPOT, *n* (%)	
Triple therapy (steroids + MMF + CNI-like)	33 (66)
Dual therapy	17 (34)
Tacrolimus trough at ELISPOT, ng/ml^a^	6.2 (5.0–6.9)
MMF dose at ELISPOT, g/d	1.0 (1.0–1.0)
Structural ELISPOT score (BKV_struct), SFC/10^6^ PBMC	100.0 (46.0–317.8)
Regulatory ELISPOT score (BKV_reg), SFC/10^6^ PBMC	42.5 (2.5–112.1)
Total lymphocyte count at ELISPOT, cells/μl	354 (258–706)
CD4+ T-cell count at ELISPOT, cells/μl	223 (95–475)
CD8+ T-cell count at ELISPOT, cells/μl	142 (83–235)
Biopsy-proven BK virus nephropathy, *n* (%)	9 (18)
Deep IS reduction (CNI switch/withdrawal), *n* (%)	13 (26)
Viral clearance (2 negative BK PCRs), *n* (%)	37 (74)
Early clearance (<90 d after ELISPOT), *n* (%)	8 (16)

cPRA, calculated panel reactive antibody; CNI, calcineurin inhibitor; ELISPOT, enzyme-linked immunosorbent spot.

Induction therapy consisted of basiliximab in 26 patients (52%) and thymoglobulin in 24 (48%). Three patients (6%) had experienced a treated rejection episode before the onset of BKPyV DNAemia. The median time from transplantation to first BK viremia was 3.0 months (1.9–4.0). The median time from first BK viremia to ELISPOT was 17.4 days (11.1–35.2). An initial reduction of MMF had been implemented in 23 of 50 patients (46%). The median BK viral load at ELISPOT was 3.86 log_10_ copies/ml (3.46–4.50). The median maximum BK viral load (CV_max) during the episode was 4.63 log_10_ copies/ml (4.01–5.27). The structural ELISPOT score (BKV_struct) correlated very weakly with CV_max (Pearson r ≈ –0.13).

At ELISPOT assessment, 33 patients (66%) received triple therapy and 17 (34%) dual therapy. Detailed IS drug exposures at the time of ELISPOT are provided in Supplementary Tables S1 and S2. The majority of patients (64%) were maintained on a standard triple regimen, whereas 26% were on dual therapy. Among patients receiving tacrolimus, the median trough level was 6.2 ng/ml (5.0–6.9). The median MMF dose was 1.0 g/d (1.0–1.0). The structural ELISPOT score (BKV_struct) varied widely, with a median of 100 SFC/10^6^ PBMC (46–317.8; range 0–1931). The regulatory score (BKV_reg) was lower (median 42.5 SFC/10^6^ PBMC [2.5–112.1]). At the time of ELISPOT, median total lymphocyte count was 354 cells/μl (258–706), with median CD4 and CD8 counts of 223 (95–475) and 142 (83–235) cells/μl, respectively. Structural ELISPOT responses showed no meaningful correlation with total lymphocyte count (r ≈ 0.03), CD4 (r ≈ 0.08), or CD8 counts (r ≈ –0.08).

During follow-up, 13 patients (26%) required deep IS reduction, 9 (18%) developed biopsy-proven BKPyVAN, and 37 (74%) achieved viral clearance, including 8 patients (16%) within 90 days of ELISPOT.

### Structural ELISPOT Responses and Time to BK Viral Clearance

**Figure 1:**
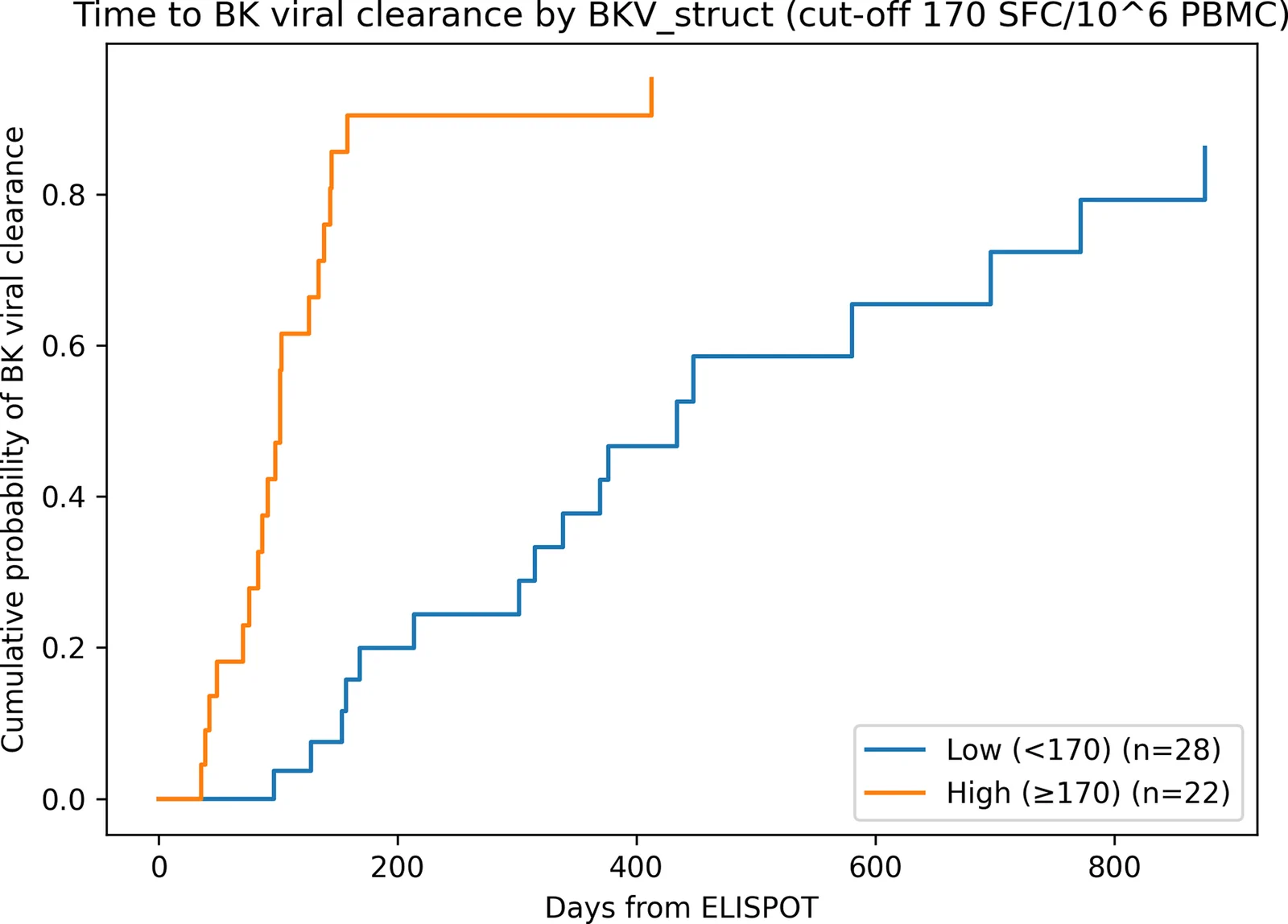
Time from ELISPOT to BK viral clearance according to structural BK ELISPOT cut-off. Kaplan–Meier curves showing the cumulative probability of achieving BK viral clearance (2 consecutive plasma BK PCR values below the lower limit of quantification) after the ELISPOT, stratified by structural ELISPOT score (BKV_struct) using an exploratory ROC-derived cut-off of 171 SFC/10^6^ PBMC. Patients with low BKV_struct (< 171 SFC/10^6^ PBMC, *n* = 28) had slower and less frequent viral clearance (17/28, 61%) with a median time to clearance of 338 days, whereas those with high BKV_struct (≥ 171 SFC/10^6^ PBMC, *n* = 22) had more frequent and faster clearance (20/22, 91%) with a median time of 100 days. ELISPOT, enzyme-linked immunosorbent spot; PBMC, peripheral blood mononuclear cells; PCR, polymerase chain reaction.

Overall, 37 of 50 patients (74%) achieved BK viral clearance with a median time from ELISPOT to clearance of 143 days (IQR 97–338). Using the exploratory threshold of 171 SFC/10^6^ PBMC, patients with low BKV_struct exhibited slower and less frequent viral clearance than those with high BKV_struct (61% vs. 91%; median time to clearance 338 vs. 100 days; Figure 1).

In univariable Cox analysis, higher BKV_struct was associated with faster viral clearance (hazard ratio: 1.20 per 100 SFC, 95% CI: 1.10–1.31; *P* < 0.0001; Table 2).

**Table 2:** Association between structural BK ELISPOT responses (BKV_struct) and clinical outcomes. Cox proportional hazards model – Time from ELISPOT to BK viral clearance

Covariate	Adjusted HR	95% CI	*P*-value
BKV_struct_100 (per +100 SFC/10^6^ PBMC)	1.28	1.15–1.42	< 0.0001
log10 BK viral load at ELISPOT	0.78	0.50–1.22	0.274
Dual vs. triple immunosuppression at ELISPOT	2.04	0.83–5.03	0.120
Age at transplantation (per yr)	1.00	0.97–1.03	0.944
Time from transplantation to first BK viremia	0.94	0.84–1.04	0.223

ELISPOT, enzyme-linked immunosorbent spot; PBMC, peripheral blood mononuclear cells.

This association remained significant after adjustment for BKPyV viral load, IS intensity, age, and time from transplantation to first BKPyV DNAemia (adjusted hazard ratio: 1.28 per 100 SFC, 95% CI: 1.15–1.42; *P* < 0.0001).

No violation of the proportional hazards assumption was observed for BKV_struct (global Schoenfeld test *P* > 0.10).

### Structural ELISPOT Responses and BKVN

**Figure 2:**
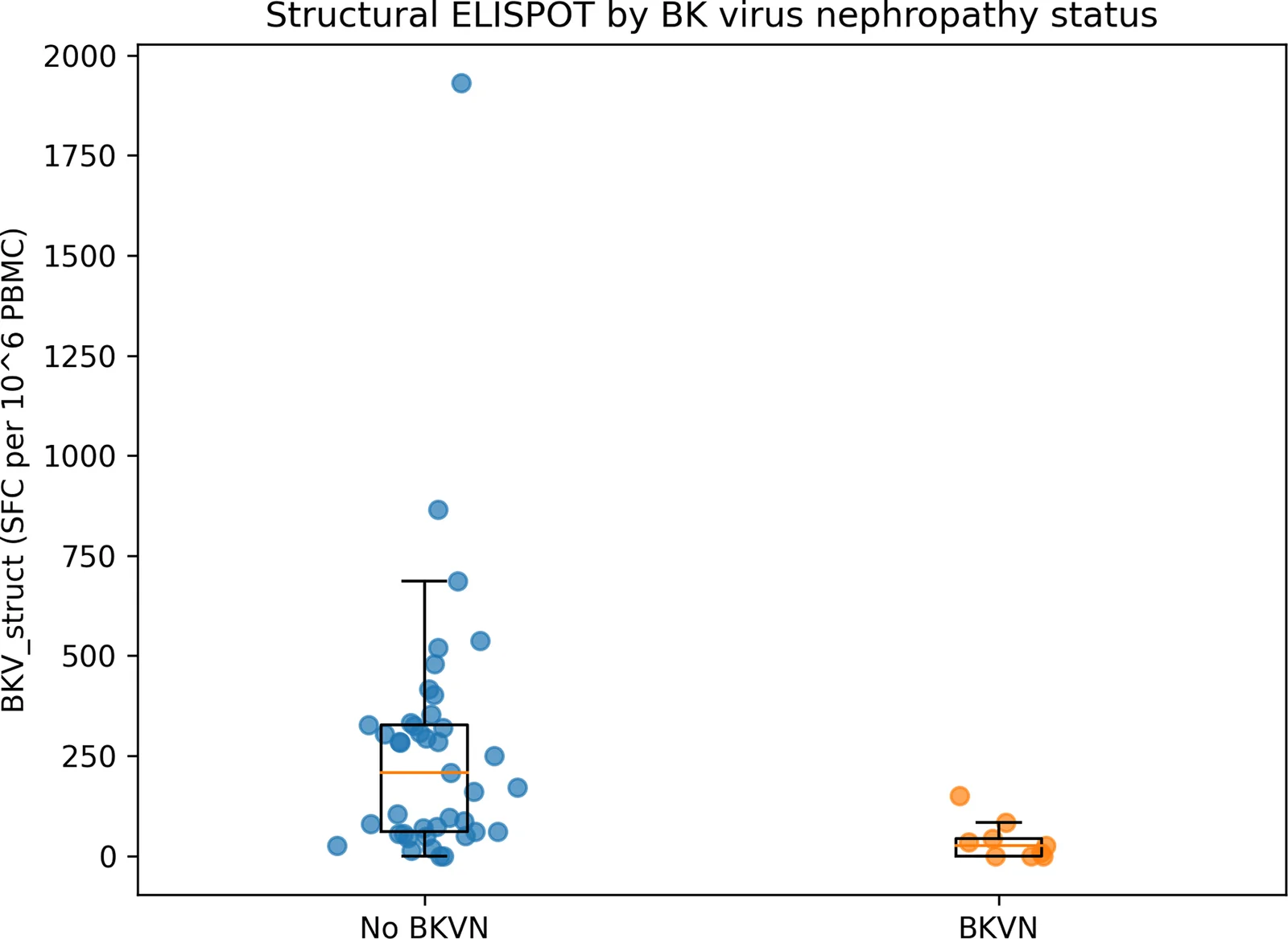
Structural BK ELISPOT responses according to biopsy-proven BK virus nephropathy. Structural ELISPOT scores (BKV_struct) in patients who did (BKVN+, *n* = 9) and did not (BKVN−, *n* = 41) develop biopsy-proven BK virus nephropathy during follow-up. Boxplots display medians, interquartile ranges and 5th–95th percentiles, with individual values overlaid as points. BKV_struct at ELISPOT was lower in patients with BKVN (median 25 [0–43] SFC/10^6^ PBMC) compared with those without BKVN (median 209 [61–328] SFC/10^6^ PBMC). BKVN, BK virus nephropathy.

Biopsy-proven BKVN occurred in 9 of 50 patients (18%). BKV_struct responses were markedly lower in patients with biopsy-proven BKPyVAN than in those without (median 25 [0–43] vs. 208.9 [61–328] SFC/10^6^ PBMC; Figure 2).

In univariable analysis, each 100-SFC increase in BKV_struct was associated with lower odds of biopsy-proven BKPyVAN (OR: 0.18, 95% CI: 0.03–0.99; *P* = 0.049).

After adjustment for BKPyV viral load and IS intensity, the association remained of similar magnitude but no longer reached statistical significance (aOR: 0.19, 95% CI: 0.03–1.20; *P* = 0.076; Table 3), likely reflecting the limited number of events.

**Table 3:** Association between structural BK ELISPOT responses (BKV_struct) and clinical outcomes. Logistic regression – Biopsy-proven BK virus–associated nephropathy

Covariate	Adjusted OR	95% CI	*P*-value
BKV_struct_100 (per +100 SFC/10^6^ PBMC)	0.19	0.03–1.20	0.076
log10 BK viral load at ELISPOT	1.55	0.56–4.24	0.397
Dual vs. triple immunosuppression at ELISPOT	0.46	0.07–2.99	0.417

ELISPOT, enzyme-linked immunosorbent spot; PBMC, peripheral blood mononuclear cells.

### Structural ELISPOT responses and depth of IS reduction

**Figure 3:**
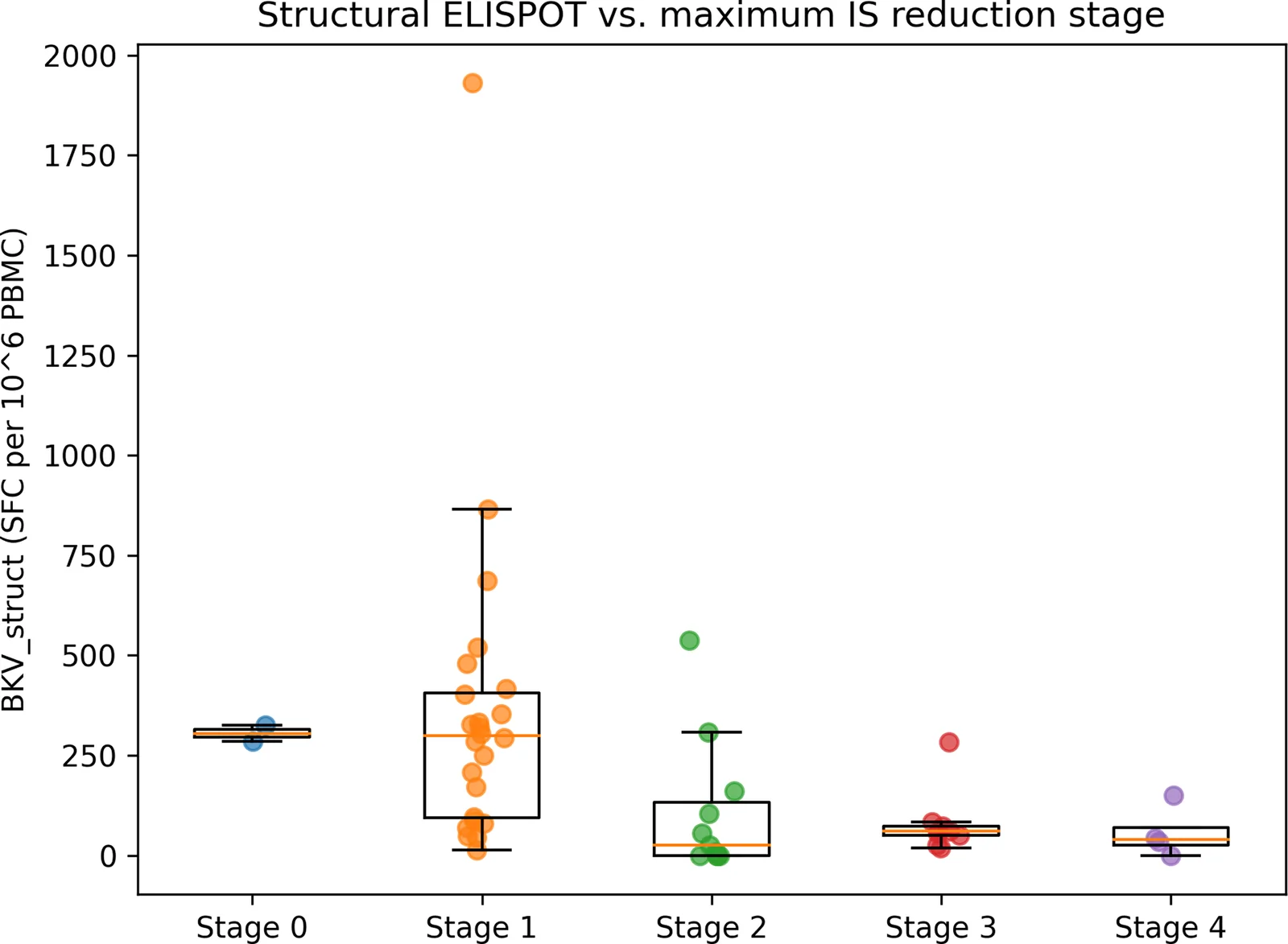
Structural BK ELISPOT responses and maximum depth of immunosuppression reduction. Distribution of structural ELISPOT scores (BKV_struct) according to the maximum immunosuppression reduction stage (IS_stage) implemented during BK management: stage 0 (no reduction), stage 1 (dose reduction and/or mammalian target of rapamycin switch only), stage 2 (MMF discontinuation without CNI switch/withdrawal), stage 3 (tacrolimus discontinuation with switch to cyclosporine), and stage 4 (CNI withdrawal with corticosteroid monotherapy). Boxplots show medians, interquartile ranges and 5th–95th percentiles; individual patient values are overlaid as jittered points. Overall, 13/50 patients (26%) required deep immunosuppression reduction (IS_stage ≥3). Patients who ultimately required deep immunosuppression reduction had markedly lower BKV_struct values at ELISPOT.

The distribution of BKV_struct according to IS reduction stage is shown in Figure 3.

In multivariable logistic regression analyses (Table 4), adjusted for BKPyV viral load, IS intensity, age, time from transplantation to first BKPyV DNAemia, and serum creatinine at ELISPOT, higher BKV_struct was independently associated with a reduced need for deep IS reduction (aOR: 0.34 per 100 SFC, 95% CI: 0.14–0.81; *P* = 0.018).

**Table 4:** Association between structural BK ELISPOT responses (BKV_struct) and clinical outcomes. Logistic regression – Deep immunosuppression reduction (IS stage ≥ 3)

Covariate	Adjusted OR	95% CI	*P*-value
BKV_struct_100 (per +100 SFC/10^6^ PBMC)	0.34	0.14–0.83	0.018
log10 BK viral load at ELISPOT	2.13	0.64–7.07	0.217
Dual vs. triple immunosuppression at ELISPOT	0.37	0.04–3.28	0.373
Age at transplantation (per year)	0.96	0.90–1.03	0.238
Time from transplantation to first BK viremia	1.46	1.00–2.13	0.049
Serum creatinine at ELISPOT (per μmol/L)	0.99	0.97–1.00	0.158

ELISPOT, enzyme-linked immunosorbent spot; PBMC, peripheral blood mononuclear cells.

This association remained significant after additional adjustment for tacrolimus trough level and MMF dose at ELISPOT (aOR: 0.50, 95% CI: 0.26–0.96; *P* = 0.036), whereas neither variable was independently associated with the outcome.

### ROC Analyses and Exploratory Structural ELISPOT Thresholds

**Figure 4:**
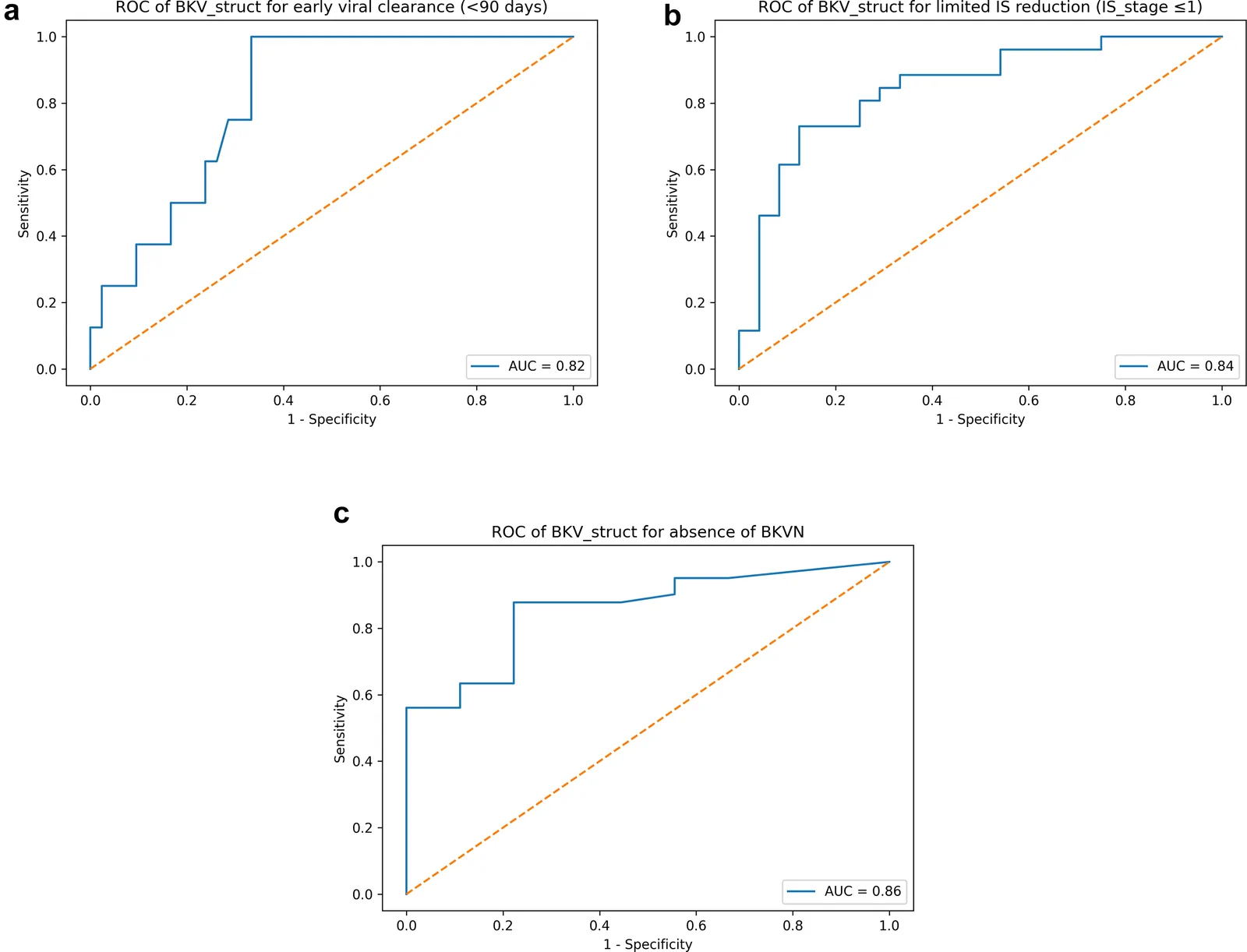
Predictive performance of structural BK ELISPOT responses for BK-related outcomes. Receiver operating characteristic (ROC) curves for the structural ELISPOT score (BKV_struct) to predict three clinically relevant outcomes: (a) early viral clearance within 90 days after ELISPOT (8/50 patients), (b) avoidance of deep immunosuppression reduction (IS_stage ≤ 2; 37/50 patients), and (c) absence of biopsy-proven BK virus nephropathy (BKVN; 41/50 patients). The area under the curve (AUC) for BKV_struct was 0.82 for early clearance, 0.84 for avoidance of major immunosuppression reduction, and 0.86 for absence of biopsy-proven BKVN. Exploratory optimal cut-offs derived from the Youden index were: 171 SFC/10^6^ PBMC for early clearance (sensitivity 100%, specificity 67%; panel a), 171 SFC/10^6^ PBMC for avoidance of major immunosuppression reduction (sensitivity 73%, specificity 88%; panel b) and 45 SFC/10^6^ PBMC for absence of biopsy-proven BKVN (sensitivity 88%, specificity 78%; panel c). Together, these ROC analyses support a high-risk profile at very low BKV_struct values (e.g., < 50 SFC/10^6^ PBMC) and a low-risk profile at higher values (e.g., ≥ 170–200 SFC/10^6^ PBMC). These thresholds are exploratory and require external validation before being used for clinical decision-making.

ROC analyses showed good discrimination of BKV_struct for several clinically relevant outcomes (Figure 4). Areas under the curves were 0.82 for early viral clearance (< 90 days), 0.84 for avoidance of deep IS reduction, and 0.86 for absence of biopsy-proven BKPyVAN. Exploratory optimal thresholds were approximately 171 SFC/10^6^ PBMC for early clearance and avoidance of deep IS reduction, and 45 SFC/10^6^ PBMC for absence of biopsy-proven BKPyVAN.

### Kidney Function Outcomes

Median serum creatinine at the time of ELISPOT was 139.5 μmol/l [109–174] and 142.0 μmol/l [119–226] at the end of follow-up. Overall, the median change in creatinine (Δcreatinine) was modest (+4.5 μmol/l [−11.0 to 25.8]). Patients with low BKV_struct (<171 SFC/10^6^ PBMC) exhibited less favorable creatinine trajectories than those with higher responses (+12 [−3 to 47] vs. −6 [−14.5 to 11] μmol/l). Patients with biopsy-proven BKPyVAN experienced a marked increase in creatinine compared with those without BKPyVAN (+116 [26–179] vs. +2 [−11 to 15] μmol/l).

### Regulatory Responses (BKV_reg)

In secondary analyses, BKV_reg (large T antigen + small T antigen) showed weaker and less consistent associations with outcomes. Although modest associations with viral clearance were observed, BKV_reg was not independently associated with biopsy-proven BKPyVAN or deep IS reduction in multivariable analyses (data not shown).

## Discussion

In this study, BKPyV-specific T-cell immunity assessed by IFN-γ ELISPOT shortly after first BKPyV DNAemia was strongly associated with virological and clinical trajectories in kidney transplant recipients. ELISPOT was performed early after first viremia under real-world management, and its associations with outcomes persisted after adjustment for IS intensity, tacrolimus level, and MMF dose.

Current management of BKPyV infection relies almost exclusively on virological monitoring and stepwise reduction of maintenance IS, as codified in international guidelines.^7^ Despite this strategy, viral trajectories remain heterogeneous, rejection may occur following IS reduction^3^^,^^4^^,^^6^^,^^10^ and adjunctive therapeutic approaches have not consistently improved outcomes, as recently illustrated by the BKEVER randomized trial.^11^ These observations suggest that virological and pharmacological approaches alone may not fully explain interindividual differences in BKPyV control, highlighting the potential contribution of host antiviral immune competence.

BKPyV-specific cellular immunity has been recognized for more than 2 decades as the central determinant of viral control after kidney transplantation. Early mechanistic studies demonstrated that IS drugs directly inhibit BKPyV-specific T-cell responses, establishing impaired virus-specific cellular immunity as the primary biological substrate of BKPyV replication rather than viral pathogenicity per se.^12^ On this concept, Binggeli *et al.*^13^ provided the first clinical evidence that IFN-γ–producing T-cell responses directed against BKPyV VP1 and large T antigen closely correlate with viral kinetics, with quantitatively dominant VP1-specific responses observed in patients with resolving infection. Subsequent longitudinal studies by Schachtner and colleagues further refined this model by demonstrating that the kinetics of BKPyV-specific T-cell recovery—particularly responses directed against structural capsid antigens (VP1–3)—predict recovery from BKPyVAN and precede viral clearance, whereas regulatory antigen-specific responses are more heterogeneous and less tightly linked to outcome.^14^, ^15^, ^16^ Importantly, qualitative analyses revealed that effective viral control is associated with functionally competent, polyfunctional effector T cells rather than mere antigen recognition, underscoring the biological relevance of functional immune readouts.^17^ A recent systematic review and meta-analysis confirmed that BKPyV-specific ELISPOT responses are consistently associated with viral resolution across studies, while highlighting the need for clinically standardized assays and meaningful end points.^18^

Humoral immunity has emerged as an important complementary component of BKPyV host defense, although its role appears distinct from that of cellular immunity. Neutralizing antibodies directed against BKPyV capsid proteins, particularly VP1, have been associated with a lower risk of post-transplant replication and BKPyVAN,^19^, ^20^, ^21^, ^22^, ^23^ These observations also provided the rationale for antibody-based approaches, including i.v. Ig administration, supported by in vitro neutralizing activity and preliminary clinical studies.^24^, ^25^, ^26^, ^27^, ^28^ However, humoral immunity alone appears insufficient to control established infection, as longitudinal studies integrating cellular and humoral monitoring have shown that BKPyV-specific IgG responses frequently increase during active replication and do not reliably distinguish resolving from persistent infection, whereas BKPyV-specific cellular responses do.^14^, ^15^, ^16^

The interpretation of structural antigen responses may also be influenced by the characteristics of the ELISPOT platform used in this study. Because overlapping 15mer peptide pools preferentially stimulate CD4+ T-cell responses,^29^ the observed associations with VP1–VP3 responses may reflect broader antiviral immune competence rather than isolated effector T-cell activity. Previous studies have reported correlations between BKPyV-specific CD4+ T-cell responses and anti-VP1 antibody levels.^30^ Recent data from the Swiss Transplant Cohort suggest that higher anti-VP1 antibody levels at transplantation are associated with subsequent clearance of BKPyV DNAemia, potentially reflecting pre-existing antiviral immune competence.^31^^,^^32^ Anti-Large T antigen antibody responses also appear later during infection and have been associated with viral clearance.^33^ Because BKPyV-specific antibody measurements were not available in our cohort, we could not directly evaluate these interactions. Future studies integrating cellular and humoral immune monitoring may provide a more comprehensive understanding of the mechanisms underlying BKPyV control. Because kidney biopsies were performed according to clinical indication rather than protocol, some cases of BKPyVAN may have remained undetected. Accordingly, comparisons should be interpreted as biopsy-proven BKPyVAN versus no biopsy-proven BKPyVAN, rather than true presence versus absence of BKPyVAN.

From a clinical perspective, our findings do not support replacing serial BKPyV PCR monitoring with immune monitoring. BKPyV-specific ELISPOT responses may provide complementary information regarding the host's ability to control viral replication beyond viral burden alone. Among all outcomes evaluated, viral clearance within 3 months may be the most clinically relevant end point, as early viral control is a major objective of current BKPyV management strategies. In our cohort, higher structural BKPyV-specific ELISPOT responses were strongly associated with early viral clearance, supporting the hypothesis that preserved antiviral immune competence contributes to rapid recovery of viral control following IS reduction. Patients with stronger structural responses may represent a lower-risk subgroup, whereas weak or absent responses could identify recipients requiring closer surveillance. Importantly, not all patients with high ELISPOT responses achieved rapid viral clearance, suggesting that BKPyV control remains multifactorial. Such an immune-stratified approach remains hypothetical and should be evaluated prospectively before being incorporated into routine clinical practice. A single ELISPOT measurement should not be interpreted as a deterministic predictor of viral clearance occurring several months later. Rather, it may identify an early immunological profile associated with subsequent viral control under standard management. This interpretation is consistent with previous reports^34^ showing that a single step of IS reduction may be sufficient in a substantial proportion of patients with BKPyV replication, suggesting that some recipients retain sufficient antiviral immune competence to regain control after limited therapeutic intervention. Serial BKPyV PCR monitoring therefore remains essential, and ELISPOT should be considered a complementary immune biomarker rather than a stand-alone predictive test.

Several limitations of this study should be acknowledged. First, its retrospective and single-center design limits causal inference and generalizability, and the sample size—particularly for biopsy-proven BKPyVAN—restricted the complexity of multivariable modeling and the precision of threshold estimation. Second, BKPyV-specific immune monitoring was performed using a locally developed ELISPOT assay. Although this assay was based on well-established methodological principles and used antigenic targets widely reported in the literature, the lack of external standardization may limit direct comparability with other platforms. However, the early timing of ELISPOT assessment represents a deliberate strength of the study, as it captures antiviral immune competence at the moment when clinical management decisions are initiated in routine practice. Serial BKPyV-specific ELISPOT measurements were not systematically performed, precluding assessment of immune-response kinetics over time, and spontaneous viral clearance could not be reliably distinguished from clearance following therapeutic IS reduction. Nearly half of the patients had already undergone an initial reduction of mycophenolate before ELISPOT testing, indicating that ELISPOT measurements cannot be considered strictly baseline assessments and may have been influenced by early therapeutic interventions. Future prospective studies should assess BKPyV-specific immunity before therapeutic intervention. Similarly, ROC-derived thresholds should be considered exploratory and hypothesis-generating only, and require validation in independent cohorts before any clinical application. Although IFNγ ELISPOT provides a functional readout of BKPyV-specific cellular immunity, it does not identify the phenotype of the IFNγ-producing cells. We did not perform complementary phenotypic analyses to identify the cellular sources of IFNγ production. Kidney function outcomes should nevertheless be interpreted with caution, as changes in serum creatinine may reflect multiple competing processes, including BKPyVAN, rejection, and modifications of maintenance IS. Consequently, the observed association between BKPyV-specific cellular immunity and kidney function evolution should be considered exploratory and hypothesis-generating. Finally, IS reduction was not protocolized and may have been influenced by clinical judgment, introducing potential confounding by indication; nevertheless, this real-world approach enhances the external validity of our findings.

In conclusion, early BKPyV-specific cellular immune responses measured by IFNγ ELISPOT were consistently associated with viral clearance kinetics and the intensity of IS reduction following BKPyV DNAemia. These findings support the potential role of immune monitoring as a complementary tool for risk stratification in kidney transplant recipients with BKPyV replication. Given the observational design, limited sample size, and absence of external validation, these results should be considered hypothesis-generating and require confirmation in prospective multicenter studies.

## Disclosure

All the authors declared no competing interests.

## Supplementary Material

Supplementary file (PDF) **Table S1.** Immunosuppressive drug exposure at the time of ELISPOT (*N* = 50). **Table S2.** Immunosuppressive regimen combinations at ELISPOT (*N* = 50). STROBE Checklist

## Footnotes

Supplementary file (PDF)
**Table S1.** Immunosuppressive drug exposure at the time of ELISPOT (*N* = 50). **Table S2.** Immunosuppressive regimen combinations at ELISPOT (*N* = 50). STROBE Checklist.
